# Independent and Synergistic Effects of Knocking out Two ABC Transporter Genes on Resistance to *Bacillus thuringiensis* Toxins Cry1Ac and Cry1Fa in Diamondback Moth

**DOI:** 10.3390/toxins13010009

**Published:** 2020-12-24

**Authors:** Shan Zhao, Dong Jiang, Falong Wang, Yihua Yang, Bruce E. Tabashnik, Yidong Wu

**Affiliations:** 1 College of Plant Protection, Nanjing Agricultural University, Nanjing 210095, China; zhaoshan2017202031@163.com (S.Z.); jiangdong0302@163.com (D.J.); wangfalong0703@163.com (F.W.); yhyang@njau.edu.cn (Y.Y.); 2 Department of Entomology, University of Arizona, Tucson, AZ 85721, USA; brucet@cals.arizona.edu

**Keywords:** *Plutella xylostella*, *ABCC2*, *ABCC3*, CRISPR/Cas9, *Bacillus thuringiensis*, resistance, Cry1Ac, Cry1Fa

## Abstract

Insecticidal proteins from *Bacillus thuringiensis* (Bt) are used widely in sprays and transgenic crops to control insect pests. However, evolution of resistance by pests can reduce the efficacy of Bt toxins. Here we analyzed resistance to Bt toxins Cry1Ac and Cry1Fa in the diamondback moth (*Plutella xylostella*), one of the world’s most destructive pests of vegetable crops. We used CRISPR/Cas9 gene editing to create strains with knockouts of the ATP-binding cassette (ABC) transporter genes *PxABCC2*, *PxABCC3*, or both. Bioassay results show that knocking out either gene alone caused at most 2.9-fold resistance but knocking out both caused >10,320-fold resistance to Cry1Ac and 380-fold resistance to Cry1Fa. Cry1Ac resistance in the double knockout strain was recessive and genetically linked with the *PxABCC2/PxABCC3* loci. The results provide insight into the mechanism of cross-resistance to Cry1Fa in diamondback moth. They also confirm previous work with this pest showing that mutations disrupting both genes cause higher resistance to Cry1Ac than mutations affecting either *PxABCC2* or *PxABCC3* alone. Together with previous work, the results here highlight the value of using single and multiple gene knockouts to better understand the independent and synergistic effects of putative Bt toxin receptors on resistance to Bt toxins.

## 1. Introduction

Insecticidal proteins from the bacterium *Bacillus thuringiensis* (Bt) have been used widely in sprays since the 1920s and in transgenic crops since 1996 to control some key insect pests [1]. Global planting of transgenic Bt crops increased from 1.1 million hectares in 1996 to 104 million hectares in 2018 [2]. Extensive use of Bt toxins has caused evolution of resistance by some target pests. Practical resistance to Bt toxins used in sprays and transgenic crops has been documented in some populations of at least 11 insect species [3,4,5,6,7]. Understanding the mechanism of resistance to Bt toxins and identifying Bt resistance genes can help to develop effective monitoring methods and management strategies for delaying evolution of resistance.

Here we focus on resistance to Bt toxins in the diamondback moth (*Plutella xylostella*), one of the most destructive pests of cruciferous vegetables worldwide [8]. This pest causes economic losses up to 5 billion dollars annually [9] and has evolved resistance to all major classes of insecticides [3]. In response to repeated exposure to sprays containing Bt toxins, it was the first pest with documented field-evolved resistance to Bt toxins [10]. Transgenic Bt host plants of this pest have been produced for experimental purposes, but are not commercially available [3].

In diamondback moth, as in many other lepidopteran pests, resistance to widely used crystalline (Cry) toxins from Bt is associated with mutations disrupting ATP-binding cassette (ABC) transporter proteins [11,12,13,14,15,16]. A naturally occurring 30-bp deletion in the gene *PxABCC2* expected to yield a truncated ABC transporter protein is genetically linked with resistance to Cry1Ac in a field-derived strain of diamondback moth from Hawaii [17,18]. Two recent studies directly tested the role of *PxABCC2* and the adjacent gene *PxABCC3* in resistance to Cry1Ac by using CRISPR/Cas9 gene editing to introduce frameshift mutations expected to produce truncated proteins [15,16]. Guo et al. [15] reported resistance to Cry1Ac was 724-fold for a knockout of *PxABCC2* alone and 413-fold for a knockout of *PxABCC3* alone. By contrast, Liu et al. [16] found that resistance to Cry1Ac caused by CRISPR editing was less than 4-fold in six strains with various knockouts of either *PxABCC2* or *PxABCC3* alone and >8000-fold in three strains with both genes knocked out. Further investigation of the roles of *PxABCC2* and *PxABCC3* is warranted to clarify the effects of single versus double knockouts on resistance to Cry1Ac and to determine the effects on resistance to other Bt toxins.

Here we used CRISPR gene editing to test the effects of knockouts of *PxABCC2*, *PxABCC3*, or both on diamondback moth resistance to Cry1Ac and Cry1Fa. Rather than introducing frameshift mutations as in previous work, our CRISPR editing deleted large portions of each gene. We found that the single gene knockouts had little or no effect, whereas the double knockout caused >10,320-fold resistance to Cry1Ac and 380-fold resistance to Cry1Fa. These results identify mutations in *PxABCC2* and *PxABCC3* as potential contributors to previously observed cross-resistance between Cry1A toxins and Cry1Fa. They also highlight the value of using single and multiple gene knockouts to better understand the independent and synergistic effects of putative Bt toxin receptors on resistance to Bt toxins.

## 2. Results

### 2.1. CRISPR-Mediated Single Knockouts of PxABCC2 or PxABCC3

**Figure 1:**
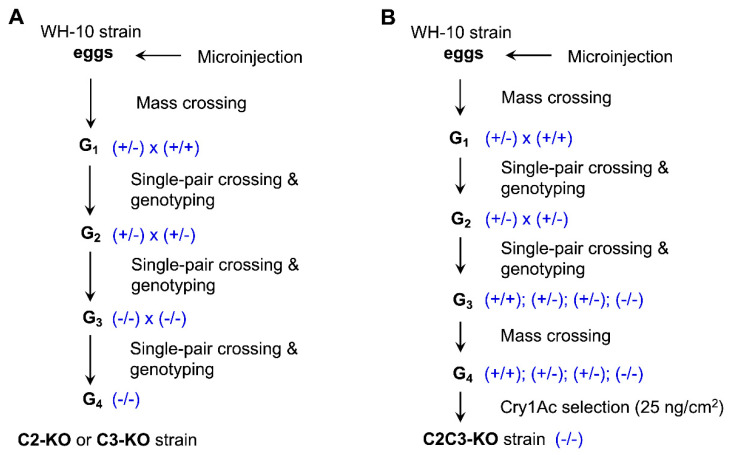
Crossing and selection for establishing homozygous knockout strains of *P. xylostella*. (**A**) Single knockout strains of *PxABCC2* (C2-KO) or *PxABCC3* (C3-KO). (**B**) Double knockout strain of *PxABCC2* and *PxABCC3* (C2C3-KO).

**Figure 2:**
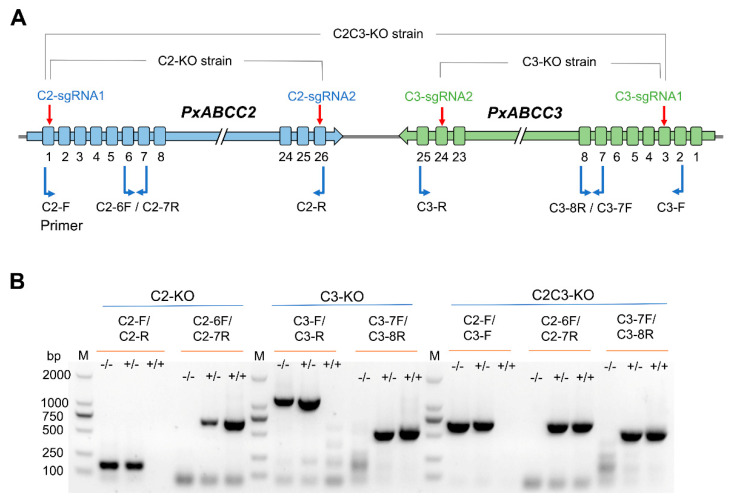
CRISPR/Cas9-mediated knockout of *PxABCC2*, *PxABCC3*, or both. (**A**) The genomic structure of *PxABCC2* and *PxABCC3*, and positions of four sgRNAs for gene editing and four primer pairs for allele-specific PCR detection. (**B**) Genotyping of individual *P. xylostella* as missing *PxABCC2*, *PxABCC3*, or both based on banding patterns from allele-specific PCR products.

**Figure 3:**
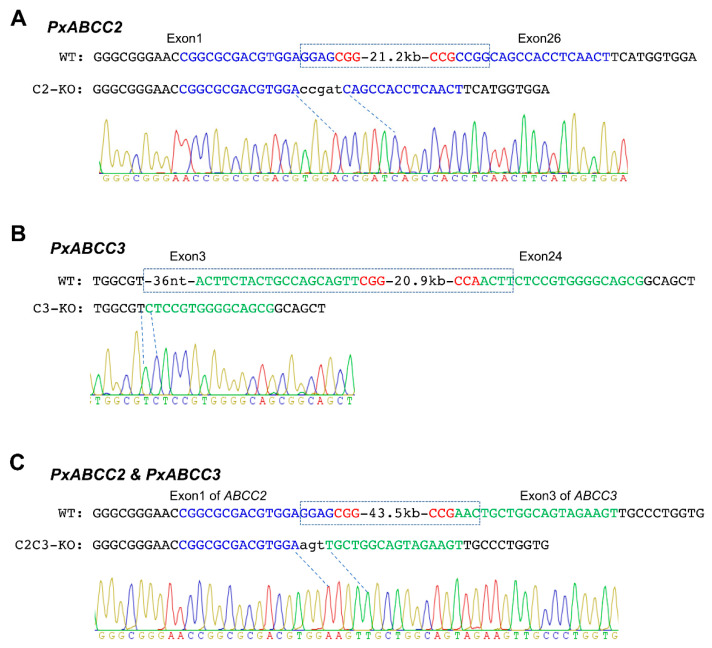
Sequences of the wild type (WT) and knockouts of *PxABCC2* and *PxABCC3* flanking the target sites of dual sgRNAs for introducing deletion mutations. (**A**) Knockout strain of *PxABCC2* (C2-KO). (**B**) Knockout strain of *PxABCC3* (C3-KO). (**C**) Double knockout strain of *PxABCC2* and *PxABCC3* (C2C3-KO). Boxes indicate the deleted genomic DNA fragments. Target sites of sgRNAs are highlighted in blue (*PxABCC2*) and green (*PxABCC3*).

To knockout *PxABCC2*, we injected C2-sgRNA1 targeting exon 1 and C2-sgRNA2 targeting exon 26 with Cas9 into 450 eggs (G_0_) of the susceptible strain WH-10 (Figure 1A and Figure 2A). Next we obtained the progeny (G_1_) from a mass cross with 85 G_0_ moths. We used 12 single-pair crosses of G_1_ moths to produce G_2_ eggs. After G_2_ eggs were collected, we genotyped the 24 G_1_ parents for the expected CRISPR-mediated deletion mutation (21.2 kb, Figure 3A) with PCR amplification and confirmed by direct sequencing of the PCR product. Although 23 of these 24 G_1_ parents lacked the 21.2 kb deletion, one was heterozygous for this deletion. From the single-pair G_2_ family that had this one parent heterozygous for the 21.2 kb deletion, we obtained the progeny (G_3_) from 24 single-pair crosses. After G_3_ eggs were collected, 48 G_2_ parents were genotyped. Seven single-pair families had both parents heterozygous for the 21.2 kb deletion. We pooled the G_3_ individuals from these seven single-pair families and set up 24 single-pair crosses to produce G_4_. Progeny from the two single-pair G_4_ families with both parents homozygous for the 21.2 kb deletion were pooled to generate the *PxABCC2* knockout strain named C2-KO.

We used an analogous process to create the *PxABCC3* knockout strain named C3-KO. We injected C3-sgRNA1 targeting exon 3 and C3-sgRNA2 targeting exon 24 co-injected with Cas9 into 407 eggs from the susceptible strain WH-10 (Figure 1A and Figure 2A). Next we obtained the progeny (G_1_) from a mass cross with 37 G_0_ moths. We used 24 single-pair crosses of G_1_ moths to produce G_2_ eggs. After G_2_ eggs were collected, we genotyped the 48 G_1_ parents with PCR amplification and confirmed by direct sequencing of the PCR product. Although 45 of these 48 G_1_ parents lacked the CRISPR-mediated deletion mutation (20.9 kb, Figure 3B), one parent from each of three single-pair crosses was heterozygous for the 20.9 kb deletion. From the three single-pair G_2_ families that each had one parent heterozygous for the deletion mutation, we obtained the progeny (G_3_) from 72 single-pair crosses. After G_3_ eggs were collected, the 144 G_2_ parents were genotyped. Seven single-pair families were identified with both parents heterozygous for the 20.9 kb deletion. We pooled the G_3_ individuals from these seven single-pair families and set up 36 single-pair crosses to produce G_4_. Progeny from the two single-pair G_4_ families with both parents homozygous for the 20.9 kb deletion were pooled to generate the *PxABCC3* knockout strain named C3-KO.

### 2.2. CRISPR-Mediated Double Knockout of PxABCC2 and PxABCC3

To knockout both *PxABCC2* and *PxABCC3*, we injected C2-sgRNA1 targeting exon 1 of *PxABCC2* and C3-sgRNA1 targeting exon 3 of *PxABCC3* with Cas9 into 325 eggs from WH-10 (Figure 1B and Figure 2A). We used a mass cross with 129 G_0_ moths to produce G_1_. A total of 96 single pairs of G_1_ were set up to produce G_2_. After G_2_ eggs were collected, 192 G_1_ parents were genotyped with PCR amplification for the expected 43.5 kb deletion (Figure 3C) and then confirmed by direct sequencing of the PCR product. We found one parent from each of two G_1_ single-pair crosses was heterozygous for the deletion mutation. We set up eight single-pair crosses between G_2_ moths from these two single-pair families. After G_3_ eggs were collected, 16 G_2_ parents were genotyped. Two single-pair families had both parents heterozygous for the 43.5 kb deletion. Progeny (G_3_) from these two single-pair families were pooled and mass crossed to produce G_4_ progeny.

Of 536 G_4_ larvae reared on diet treated with 25 ng Cry1Ac per cm^2^ diet, 95 survived (17.7%). All 27 randomly selected survivors that we genotyped were homozygous for the 43.5 kb deletion. By contrast, for 24 G_4_ larvae reared on untreated diet, only seven were homozygous for the 43.5 kb deletion, eight were heterozygotes, and nine were homozygous wild type. The proportion of larvae homozygous for the double knockout was significantly higher for the larvae from treated diet (1.0) than larvae from untreated diet (0.29) (Fisher’s exact test, *p* = 2 × 10^−8^). This indicates strong genetic linkage between the double knockout and resistance to Cry1Ac. We pooled the 68 G_4_ survivors that were not genotyped and allowed them to mate to establish the double knockout strain of *PxABCC2* and *PxABCC3* named C2C3-KO. From the G_5_ progeny produced by these 68 G_4_ survivors, we randomly sampled and genotyped 24 larvae. All 24 were homozygous for the 43.5 kb deletion indicating the double knockout of *PxABCC2* and *PxABCC3*.

### 2.3. Responses of Knockout Strains to Bt Toxins Cry1Ac and Cry1Fa

In diet overlay bioassays with Cry1Ac, the concentration of toxin lethal to 50% of larvae (LC_50_) was significantly higher for each knockout strain than the parent susceptible strain WH-10, based on the conservative criterion of no overlap of the 95% fiducial limits (Table 1). The resistance ratio, which is the LC_50_ for a knockout strain divided by the LC_50_ for WH-10, was 2.9 for C2-KO, 2.1 for C3-KO, and >10,320 for C2C3-KO (Table 1). The LC_50_ of Cry1Ac for either C2-KO or C3-KO was significantly higher than that of WH-10.

With Cry1Fa, the resistance ratio was 1.0 for C2-KO, 0.7 for C3-KO, and 380 for C2C3-KO (Table 1). Relative to the LC_50_ of Cry1Fa for WH-10, the LC_50_ did not differ significantly for C2-KO, was significantly lower for C3-KO, and significantly higher for C2C3-KO (Table 1). The LC_50_ of Cry1Fa was significantly higher for C2-KO than C3-KO. For both Cry1Ac and Cry1Fa, the LC_50_ for C2-KO was 1.4-fold higher than for C3-KO.

### 2.4. Recessive Resistance to Cry1Ac in the Double Knockout Strain C2C3-KO

On diet treated with 25 ng Cry1Ac per cm^2^ diet, survival was 0% for WH-10 and 100% for C2C3-KO (Table 2). At this concentration, survival did not differ significantly between the F_1_ progeny from C2C3-KO females and WH-10 males (3%) and the F_1_ progeny from WH-10 females and C2C3-KO males (0%) (Fisher’s exact test, *p* = 0.25, Table 2). These results indicate inheritance of resistance to Cry1Ac in C2C3-KO was autosomal and recessive, with no evidence of substantial maternal effects or sex linkage. Based on the data pooled from the two reciprocal crosses, the value of the dominance parameter *h* is 0.016, indicating almost completely recessive inheritance.

## 3. Discussion

In this study, resistance of diamondback moth to Cry1Ac caused by CRISPR-mediated deletions was >10,320-fold with both *PxABCC2* and *PxABCC3* knocked out, but only 2.9-fold with knockout of *PxABCC2* alone and 2.1-fold with knockout of *PxABCC3* alone. These results imply that both *PxABCC2* and *PxABCC3* can affect the susceptibility of diamondback to Cry1Ac, consistent with findings of two previous studies [15,16]. In addition, our results indicate that knocking out *PxABCC2* alone conferred numerically higher resistance to Cry1Ac than knocking out *PxABCC3* alone, which is also consistent with previous studies [15,16]. The mean LC_50_ of Cry1Ac for one or more strains with *PxABCC2* disrupted divided by the mean LC_50_ for one or more strains with *PxABCC3* disrupted was similar across the three studies: 1.4 here, 1.8 in Guo et al. [15], and 1.4 in Liu et al. [16]. Another common finding across the three studies is that resistance to Cry1Ac was recessive.

The results here showing much higher resistance to Cry1Ac conferred by knocking out both *PxABCC2* and *PxABCC3* than knocking out either alone parallel those of Liu et al. [16]. They reported the resistance ratio was >8000 for each of three independent strains with both genes knocked out (similar to >10,320 here for C2C3-KO) versus only a mean of 2.8 for four independent strains with only *PxABCC2* knocked out (nearly identical to 2.9 here for C2-KO here). Our results and those of Liu et al. [16] support the hypothesis that some functional redundancy in mediating toxicity of Cry1Ac occurs between *PxABCC2* and *PxABCC3*.

Liu et al. [16] also found that the Cry1S1000 strain of diamondback moth from Florida had a Cry1Ac resistance ratio >8000 and naturally occurring disruptive mutations in both *PxABCC2* and *PxABCC3* that were tightly linked with resistance to Cry1Ac. Whereas we found that knocking out *PxABCC3* yielded a resistance ratio of 2.1, they reported that knocking out this gene “surprisingly” increased susceptibility to Cry1Ac by a mean of 3.2-fold in two independent strains (range = 2.6 to 3.8-fold).

In contrast with the results here and in Liu et al. [16] showing resistance ratios less than 4 in all eight single knockout strains tested, Guo et al. [15] reported resistance ratios of 724 and 413 from single knockouts of *PxABCC2* or *PxABCC3*, respectively. The >400-fold higher resistance to Cry1Ac in the single knockout strains of Guo et al. [15] could be caused by differences between studies in the bioassay methods, the mutations introduced, and/or strains analyzed. Whereas Guo et al. [15] used leaf dip bioassays, we and Liu et al. [16] used diet overlay bioassays. Although we used activated Cry1Ac toxin, both Guo et al. [15] and Liu et al. [16] used Cry1Ac protoxin. Whereas we started bioassays with second instars, both Guo et al. [15] and Liu et al. [16] tested third instars. It is unlikely that the difference between leaf dip and diet overlay bioassays, the only consistent difference in bioassay methods between Guo et al. [15] relative to both our study and Liu et al. [16], was the sole or primary cause of the difference in results between Guo et al. [15] and the other two studies. Whereas we deleted large portions of each gene, Guo et al. [15] and Liu et al. [16] introduced frameshift mutations. However, all three studies used mutations expected to knock out the target genes. Moreover, Liu et al. [16] obtained the same results with a variety of frameshift mutations in each gene. Thus, it is unlikely the specific mutations used to knockout the genes caused the difference in results between Guo et al. [15] and the other two studies. Variation in response to Cry1Ac occurs among susceptible strains of diamondback moth [19]. However, the same susceptible strain from the United States (Geneva 88) was used by Liu et al. [16] and Guo et al. [15] (renamed DBM1Ac-S, see Guo et al. [20]), whereas we used a different susceptible strain from Wuhan, China (WH-10). Thus, the susceptible strain analyzed per se can be excluded as a primary cause of the difference between the results of Liu et al. [16] and Guo et al. [15]. We hypothesize that in the single knockout strains of Guo et al. [15], a mutation at one or more loci other than *PxABCC2* and *PxABCC3* interacted with each knockout mutation to cause the >400-fold resistance.

As far as we know, this study is the first to examine the effects of knocking out *PxABCC2, PxABCC3*, and both on diamondback resistance to Cry1Fa. Similar to our results with Cry1Ac, the resistance ratio was much higher for the double knockout strain (380 for C2C3-KO) than for either of the single knockout strains (1.0 for C2-KO and 0.7 for C3-KO). However, for each of the three knockout strains the resistance ratio was lower for Cry1Fa than Cry1Ac, implying that *PxABCC2* and *PxABCC3* are less important in mediating toxicity of Cry1Fa than Cry1Ac.

It is noteworthy that knocking out *PxABCC3* alone in this study increased susceptibility to Cry1Fa by 1.4-fold, a small but statistically significant difference. Together with the surprising finding of Liu et al. [16] noted above, this unexpected result yields a total of three strains of diamondback moth in which knocking out *PxABCC3* alone significantly increased susceptibility to a Cry1 toxin. Furthermore, knockout of *SeABCC3* in *Spodoptera exigua* increased susceptibility to Cry1Ac by 1.8-fold, to Cry1Fa by 2.2-fold, and to Cry1Ca by 2.7-fold [21]. Collectively, these results suggest that in some cases, when the gene encoding ABCC2 is not knocked out, ABCC3 may interfere with toxicity. One hypothesis is that in such cases, binding of toxin to ABCC3 reduces binding of toxin to ABCC2, but binding to ABCC3 causes less mortality than binding to ABCC2.

In related work with diamondback moth, the NO-QAGE strain from Hawaii showed >10,000-fold resistance to Cry1A toxins and >10,000 cross-resistance to Cry1Fa [22,23]. Cry1Ac resistance in NO-QAGE was tightly linked with a 30-bp deletion in exon 20 of *ABCC2* [17,18]. It would be intriguing to determine if *PxABCC3* is also disrupted in NO-QAGE. The >10,000-fold cross-resistance to Cry1Fa in NO-QAGE versus the 380-fold resistance of C2C3-KO suggests that NO-QAGE may harbor mutations at one or more loci other than *PxABCC2* and *PxABCC3* that boost resistance to Cry1Fa.

Our results and those of Liu et al. [16] for diamondback moth resistance to Cry1Ac are analogous to those of Wang et al. [24] for *H. armigera* resistance to Cry1Ac. For *H. armigera*, the Cry1Ac resistance ratio was 3.8 for a strain with knockout of only *HaABCC2*, 0.9 for a strain with a knockout of only *HaABCC3*, and >15,000 for a strain with both genes knocked out [24]. However, in *S. exigua*, knockout of *SeABCC2* alone caused >470-fold resistance to Cry1Ac and 240-fold resistance to Cry1Fa [21]. In *Ostrinia furnacalis*, knocking out *OfABCC2* alone created >300-fold resistance to Cry1Fa, but only 8.1-fold resistance to Cry1Ac [25]. In *Spodoptera frugiperda*, knockout of SfABCC2 alone conferred 118-fold resistance to Cry1Fa [26]. Mutations in *Trichoplusia ni* affecting either *TnABCC2* or *TnCad* alone caused less than 8-fold resistance to the genetically modified Bt toxin Cry1Ac-A01s, whereas knocking out both yielded 3800-fold resistance [27]. These results show that the role of ABCC2 in toxicity of Cry1Ac and Cry1Fa varies among lepidopteran species.

The results reported here and previously for diamondback moth and *H. armigera* resistance to Cry1Ac [16,24] and *T. ni* resistance to Cry1Ac-A01s [27] demonstrate that even if knocking out a gene by itself has little or no effect on susceptibility to a Bt toxin, one cannot exclude the possibility that when that gene is not knocked out, the protein it encodes interacts with other proteins to affect susceptibility. Thus, we encourage evaluation of strains with multiple knockouts, as done here and in some previous studies [16,24,27].

Field-evolved resistance is more likely if one mutation is sufficient to substantially decrease susceptibility than if mutations in two different genes are required, as reported here for *PxABCC2* and *PxABCC3* and resistance to Cry1Ac and Cry1Fa and previously for *HaABCC2* and *HaABCC3* and resistance to Cry1Ac [24]. The widespread practical resistance of diamondback moth to Cry1 toxins [3] suggests that if two mutations are required, each mutation must not have been extremely rare before field populations were exposed extensively to the Bt toxins. Whereas determining the effects of knocking out genes singly or in combinations provides direct evidence about which mutations can cause resistance, analyzing insects collected from the field is the only way to determine which mutations are actually associated with practical resistance [28,29,30,31]. In conjunction with bioassays, it may be useful to monitor the frequency of mutations disrupting *ABCC2* and *ABCC3* to better understand their practical impact in field populations of diamondback moth and other pests.

## 4. Materials and Methods

### 4.1. Insect Strains

The susceptible strain WH-10 was started with insects collected in May 2010 from Wuhan (Hubei Province, China) and maintained in the laboratory without exposure to Bt toxins or other insecticides. Larvae were reared with an artificial diet purchased from Southland Products Incorporated (Lake Village, AR, USA). Adults were supplied with 10% sugar solution. Insects were kept in an environmentally controlled rearing room at 26 ± 1 °C, 50 ± 10% relative humidity and a photoperiod of 16 h light: 8 h dark.

During June 2018 to August 2020, we created the C2-KO, C3-KO and C2C3-KO strains from WH-10 with the CRISPR/Cas9 gene editing technique and single-pair family selection (Figure 1). C2-KO strain is homozygous for a 21.2 kb deletion of the genomic fragment between exon 1 and exon 26 of *PxABCC2* (Figure 2A and Figure 3A). C3-KO is homozygous for a 20.9 kb deletion of the genomic fragment between exon 3 and exon 24 of *PxABCC3* (Figure 2A and Figure 3B). C2C3-KO is a double knockout strain that is homozygous for a 43.5 kb deletion of the genomic fragment between exon 1 of *PxABCC2* and exon 3 of *PxABCC3* (Figure 2A and Figure 3C). See the Results Section 2.1 and Section 2.2 above for additional details.

### 4.2. Bt Toxins and Bioassays

Bt activated toxins Cry1Ac and Cry1Fa used in this study were purchased from Marianne Pusztai-Carey (Case Western Reserve University, Cleveland, OH, USA).

Diet overlay bioassays were used to determine the toxicity of Cry1Ac and Cry1Fa against the susceptible WH-10 strain and the three knockout strains. A series of gradient concentrations of activated toxins were prepared by diluting stock toxin solution with 0.01 M, pH 7.4, phosphate buffered saline (PBS). Liquid artificial diet (1 mL) was dispensed into each well of a 24-well plate. After the diet cooled and solidified, 100 μL of the prepared toxin solution was applied evenly to the diet surface in each well. After the wells dried at room temperature, a second instar larva was put in each well. For each concentration, 24 to 48 larvae were treated. When mortality was scored after 5 days, larvae were considered dead if they were dead or remained second instars.

The LC_50_ (the concentration of toxin killing 50% of tested larvae) and its 95% fiducial limits for each strain were calculated by probit analysis using Statistical Product and Service Solutions (SPSS) statistics (Version 18.0. SPSS Inc.: Chicago, IL, USA).

### 4.3. Cas9 Protein and sgRNAs

TrueCut^TM^ Cas9 Protein v2 was purchased from Thermo Fisher (Shanghai, China). The sgRNA target sites of *PxABCC2* (GenBank no. KM245561) and *PxABCC3* (GenBank no. KM245562) were identified respectively using the design principle of 5′-N_18-19_NGG-3′ (underlined is the PAM sequence) (Gene structures were sketched in Figure 2A). Four sgRNAs were used in this study (C2-sgRNA1 targeting at exon 1 of *PxABCC2*: CGGCGCGACGTGGAGGAGCGG; C2-sgRNA2 targeting at exon 26 of *PxABCC2*: CCGCCGGCAGCCACCTCAACT; C3-sgRNA1 targeting at exon 3 of *PxABCC3*: ACTTCTACTGCCAGCAGTTCGG; C3-sgRNA2 targeting at exon 24 of *PxABCC3*: CCAACTTCTCCGTGGGGCAGCG). Two oligonucleotides were used to synthesize sgRNA template: a forward primer harboring the T7 promoter sequence and the target sequence (5′-TAATACGACTCACTATAGN_18-19_GTTTTAGAGCTAGAAATAGC-3′) and the universal oligonucleotide encoding the remaining sgRNA sequences (5′-AAAAGCACCGACTCGGTGCCACTTTTTCAAGTTGATAACGGACTAGCCTTATTTTAACT TGCTATTTCTAGCTCTAAAAC-3′). The fusion PCR reaction system and purification of PCR products were the same as reported by Wang et al. [32,33]. The sgRNAs were synthesized by in vitro transcription of the purified DNA templates utilizing the GeneArt^TM^ Precision gRNA Synthesis Kit (Thermo Fisher Scientific, Vilnius, Lithuania) according to the manufacturer’s instruction.

### 4.4. Embryo Microinjection

The newly laid eggs were collected from a parafilm pre-painted with radish juice, washed out with 1% sodium hypochlorite solution, and rinsed with distilled water for three times. The eggs were lined up on double-sided adhesive tape attached to a microscope slide. FemtoJet and InjectMan NI 2 microinjection systems were used to inject about 1 nL of a mixture solution of sgRNA (500 ng/μL) and Cas9 protein (100 ng/μL) into each egg. Injected eggs were placed at 25 ± 1 °C and 60 ± 10% RH until hatching.

### 4.5. Genomic DNA Extraction and Mutation Identification

Genomic DNAs of individual insects (moth or larva) were extracted with AxyPrep^TM^ DNA Extraction Kit (Axygen Biosciences, Union City, CA, USA) for genotyping purpose. The direct sequencing of PCR products was conducted by TsingKe (Nanjing, China) to detect the indel mutation types on the target sites of individual genes. During the establishment of the knockout strains, large fragment deletion mutations of the parents from single-pair families were determined by banding patterns of PCR fragments amplified with two or three pairs of specific primers of *PxABCC2* and *PxABCC3* (Figure 2B). Sequences for the specific primer pairs were summarized in Table 3.

### 4.6. Inheritance of Cry1Ac Resistance in the Double Knockout Strain

Ten pairs of adults were reciprocally mass crossed between the double knockout strain (C2C3-KO) and the susceptible WH-10 strain. Survival (%) of C2C3-KO, WH-10, and their F_1_ progeny were determined at the diagnostic concentration of Cry1Ac (25 ng/cm^2^). According to the formula of Liu and Tabashnik [34], the dominance parameter *h* was calculated as: (survival rate of F_1_ − survival rate of WH-10)/(survival rate of C2C3-KO − survival rate of WH-10). The *h* values range from 0 (completely recessive) to 1 (completely dominant).

## Figures and Tables

**Table 1:** Responses to Cry1Ac and Cry1Fa of the susceptible WH-10 strain and three knockout strains of *P. xylostella*.

Toxin	Strain	Slope ± SE	LC_50_ (95% FL ^1^) (ng/cm^2^)	RR ^2^
Cry1Ac	WH-10	2.92 ± 0.38	3.10 (2.57–3.67)	
	C2-KO	1.97 ± 0.30	8.92 (6.74–13.40)	2.9
	C3-KO	3.19 ± 0.55	6.59 (4.88–8.25)	2.1
	C2C3-KO		>32,000 ^3^	>10,320
Cry1Fa	WH-10	8.72 ± 1.17	10.48 (9.77–11.14)	
	C2-KO	5.99 ± 0.96	10.60 (9.05–11.97)	1.0
	C3-KO	7.20 ± 1.09	7.47 (6.89–8.10)	0.7
	C2C3-KO	3.91 ± 0.59	3971 (3314–4821)	380

^1^ 95% fiducial limits. ^2^ RR (resistance ratio) = LC_50_ of knockout strain divided by LC_50_ of WH-10. ^3^ No mortality was observed at 32,000 ng/cm^2^, the highest concentration tested against C2C3-KO.

**Table 2:** Inheritance of resistance to Cry1Ac in the C2C3-KO strain of *P. xylostella*.

Strain/Cross	N ^1^	Survival (%)	Dominance Value (*h*) ^2^
WH-10	48	0	
C2C3-KO	48	100	
C2C3-KO♀×WH-10♂ (F_1a_)	96	3	0.03
C2C3-KO♂×WH-10♀ (F_1b_)	96	0	0

^1^ Number of larvae treated with 25 ng of Cry1Ac per cm^2^ diet. ^2^ The dominance value (*h*) = (survival of F_1_ − survival of WH-10)/(survival of C2C3-KO − survival of WH-10). The *h* value varies from 0 (completely recessive) to 1 (completely dominant).

**Table 3:** The primer pairs used for the identification of mutation types on different target sites.

Name	Primer Sequences (5′ > 3′)	PCR Cycling Condition	Product Size (bp)
C2-F C2-R	GAGCCCGGAAAGAGTCGGAAGA TGTTGTCTCCGGTCTCCTC	95 °C 30 s 59.5 °C 30 s 72 °C 30 s	~170
C2-6F C2-7R	GGGAGATCCCCTTCCAGAAG AACTCCTGAAGTCTTTCCAATGAG	95 °C 30 s 57 °C 30 s 72 °C 60 s	~660
C3-F C3-R	GAGCCGTCGTACCCCAAGGTGTTAT TGCTTTGAAAATACGCTTCCT	95 °C 30 s 57.5 °C 30 s 72 °C 90 s	~1130
C3-7F C3-8R	GAACATCACGCTGATCCTGC GACTGATAGGACAAGGGCCG	95 °C 30 s 58.5 °C 30 s 72 °C 40 s	~530
C2-F C3-F	GAGCCCGGAAAGAGTCGGAAGA GAGCCGTCGTACCCCAAGGTGTTAT	95 °C 30 s 62 °C 30 s 72 °C 60 s	~670

## Data Availability

The data presented in this study are available in Table 1 and Table 2.
